# Targeted synthesis of zeolites from calculated interaction between zeolite structure and organic template

**DOI:** 10.1093/nsr/nwac023

**Published:** 2022-02-23

**Authors:** Qinming Wu, Huimin Luan, Feng-Shou Xiao

**Affiliations:** Key Lab of Biomass Chemical Engineering of Ministry of Education, College of Chemical and Biological Engineering, Zhejiang University, Hangzhou 310027, China; Key Lab of Applied Chemistry of Zhejiang Province, Department of Chemistry, Zhejiang University, Hangzhou 310007, China; Key Lab of Biomass Chemical Engineering of Ministry of Education, College of Chemical and Biological Engineering, Zhejiang University, Hangzhou 310027, China

**Keywords:** zeolite, organic template, targeted synthesis, interaction energy, theoretical simulation

## Abstract

Zeolites, a class of silica-based porous materials, have been widely employed in the chemical industry for uses such as sorption, separation, catalysis and ion exchange. Normally, the synthesis of zeolites is performed in the presence of organic templates via a trial-and-error route, which is labor-intensive and empirical. In recent years, theoretical simulation from the interaction between a zeolite structure and an organic template has been used to guide the synthesis of zeolites, which is time-saving. In this review, recent progress in the targeted synthesis of zeolites from interaction between a zeolite structure and an organic template are briefly outlined including the design of new templates for zeolite synthesis, preparation of zeolites with new composition, development of novel routes for zeolite synthesis, synthesis of intergrowth zeolites, generation of novel zeolite structures, control of zeolite morphology and modulation of aluminum distribution in zeolites. These targeted syntheses reveal that the minimum energy principle from the theoretical simulation is key for guiding zeolite crystallization. This review will be important for zeolite researchers for rationally synthesizing zeolites and effectively designing new zeolite structures.

## Teaser

This paper summarizes recent progresses for targeted synthesis of zeolites from interaction between zeolite structure and organic template including design of new templates for zeolite synthesis, preparation of zeolites with new composition, development of novel routes for zeolite synthesis, synthesis of intergrowth zeolites, generation of novel zeolite structures, control of zeolite morphology, and modulation of aluminum distribution in the zeolites, which reveals that the minimum energy principle from the theoretical simulation is a key for zeolite crystallization.

## INTRODUCTION

Zeolites consist of corner-sharing TO_4_ (Al, Si and P) tetrahedra as the primary building units, having well-defined micropores. In the initial stage, the artificial synthesis of zeolites such as A and X reported by Barrer and Milton was always carried out in inorganic systems. Later, the addition of organic quaternary ammonium cations and organic amines as organic templates in zeolite synthesis generates many novel zeolite structures. So far, >250 zeolite frameworks have been accepted by the Structure Commission of the International Zeolite Association and each of them has a special three-letter code [1]. Currently, zeolites have been widely used in many industrial processes such as sorption, separation, catalysis and ion-exchange processes [2–10].

Normally, the discovery of novel structure zeolites in the presence of organic templates is performed via the trial-and-error route, which is labor-intensive and empirical. To overcome this issue, theoretical simulation from the interaction between zeolite frameworks and organic templates to guide the experimental zeolite synthesis has been employed. For example, many organic templates have been theoretically designed for the rational synthesis of zeolites with new composition and structure, desirable synthesis of controllable zeolite morphology, construction of intergrowth zeolites and prediction of aluminum distribution in zeolite crystals.

In this review, we will briefly summarize recent progress in the targeted synthesis of zeolites from interaction between zeolite frameworks and organic templates. After a simple introduction, typical examples for the targeted synthesis of zeolites guided by theoretical simulation are shown. Finally, a conclusion and perspectives are given. We believe that this review will be helpful for zeolite researchers for rationally synthesizing zeolites and easily understanding zeolite crystallization.

## TARGETED SYNTHESIS OF ZEOLITES

**Figure 1.**
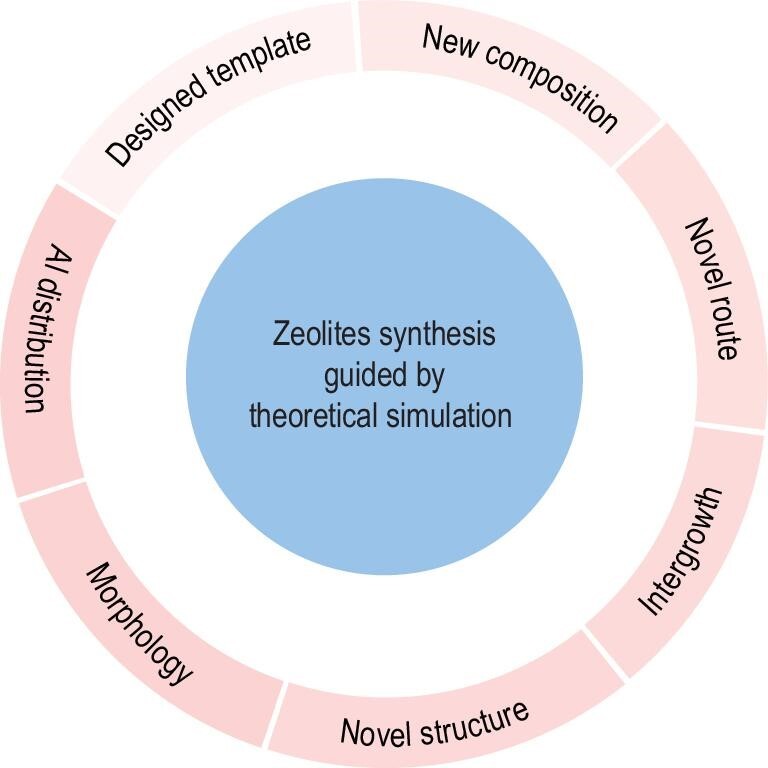
Overview of the targeted synthesis of zeolites guided by theoretical simulation.

In the past decade, great efforts have been made in synthesizing zeolites guided by energies simulated from the interaction between zeolite frameworks and organic templates. The targeted synthesis of zeolites mainly includes the design of new templates for zeolite synthesis, preparation of zeolites with new compositions, development of novel routes for zeolite synthesis, synthesis of intergrowth zeolites, generation of novel zeolite structures, control of zeolite morphology and modulation of aluminum distribution in zeolites, as shown in Fig. 1.

### Design of new organic templates for zeolite synthesis

Generally, the structure of organic templates is critical for the successful synthesis of specific zeolites. In many cases, organic templates with complex structures are costly in zeolite synthesis, which makes them very difficult to use for practical application in industrial processes. One of solutions is to discover low-cost organic templates to synthesize these zeolites assisted by theoretical simulation. This topic has been very successful in recent years and typical examples for the synthesis of zeolites such as BOG, STW, SFW, RTH and AEI structures are given in this subsection.

Boggsite as a natural zeolite is difficult to prepare artificially [11]. To solve this problem, Corma *et al.* calculated the stabilization energy between the BOG structure and organic templates, showing that the commercial *tert*-butyl-imino-tris(dimethylamino)phosphorane has the best stabilization between this organic template and Boggsite. As a result, this organic template has been successfully directed for the synthesis of Boggsite zeolite (ITQ-47, BOG structure) from a high-throughput strategy [12]. This structure was confirmed using the X-ray diffraction technique, and ^13^C and ^31^P MAS NMR techniques showed that the organic molecules were indeed positioned in the zeolite pore after the crystallization. In addition, elemental analysis of the as-synthesized product displayed similar N/C and P/N ratios to those of pure organic molecules. These results demonstrate that the *tert*-butyl-imino-tris(dimethylamino)phosphorane is an efficient organic template for directing the ITQ-47 zeolite.

**Figure 2.**
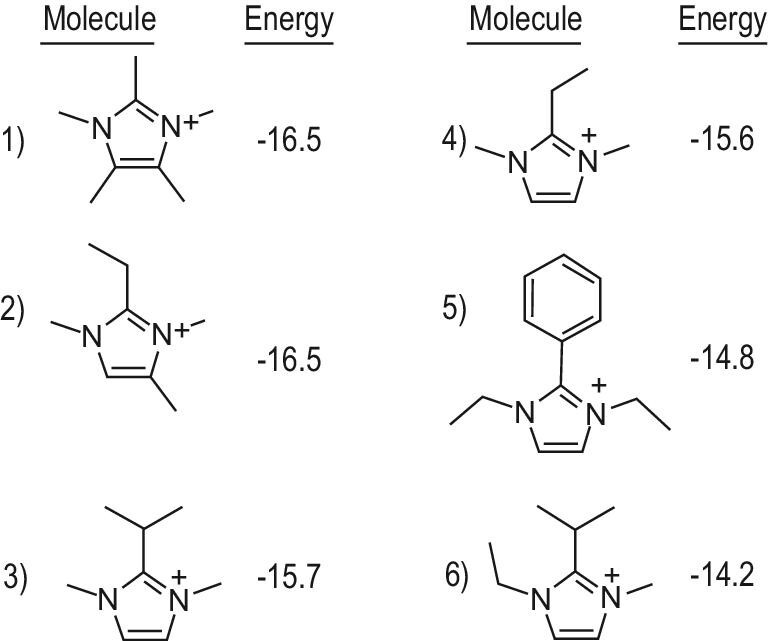
The stabilization energies (kJ (mol Si)^–1^) calculated between organic templates and a pure silica STW framework. Adapted with permission from ref. [15].

HPM-1 zeolite with STW structure is the first pure silica chiral zeolite, which is potentially important for applications [13,14]. However, the synthesis of HPM-1 zeolite is very difficult, which was only performed in the presence of 2-ethyl-1,3,4-trimethylimidazolium under a very narrow phase diagram [13]. Davis *et al.* calculated the stabilization interaction energies between a pure silica STW zeolite structure with a series of imidazole-based organic molecules, finding that pentamethylimidazolium has the same stabilization energy with the conventional organic template [15], as shown in Fig. 2. Notably, the pentamethylimidazolium has no rotational degrees of freedom compared with the conventional organic template that has many different conformations in aqueous solution. Therefore, pentamethylimidazolium might be more favorable to synthesize pure silica STW zeolite. As a result, it is directly synthesized pure silica STW zeolite in the presence of pentamethylimidazolium as an organic template.

SSZ-52 zeolite with a SFW structure has good performance in the selective catalytic reduction of NO_*x*_ with NH_3_ (NH_3_–SCR) reaction [16,17]. However, the conventional template of polycyclic quarternary ammonium [N,N-diethyl-5,8-dimethylazonium bicyclo(3.2.2.)nonane] for the synthesis of SSZ-52 zeolite is very complex and costly, which strongly hinders its wide application. Davis *et al.* investigated the interaction energies between the SSZ-52 framework with organic templates that were inexpensive and easily synthesized from commercially available reagents. They found that N-ethyl-N-(2,4,4-trimethylcyclopentyl)pyrrolidinium and N-ethyl-N-(3,3,5-trimethylcyclohexyl)pyrrolidinium with large stabilization energies (more negative) were suitable templates for the rational synthesis of SSZ-52 zeolite [18]. In order to synthesize SSZ-52 zeolite economically, the chosen organic templates should (i) be easily available raw materials, (ii) require no more than three steps for the preparation and (iii) have a high yield of organic molecules.

RTH zeolite exhibits excellent performance in methanol-to-olefin (MTO) and NH_3_–SCR reactions [19–21]. In the past decade, great progress has been made in developing different routes to synthesize RTH zeolite [19–22]. However, the crystallization rate of RTH zeolite is still slow, giving low synthesis efficiency. Xu *et al.* found that the interaction energy of the novel organic template 2,6-methyl-N-methylpyridinium cation with the RTH zeolite framework is much larger (more negative) than those of conventional templates for the synthesis of RTH zeolites such as N-ethyl-N-methyl-5,7,7-trimethyl-azonium bicyclo[4.1.1]octane cations and 1,2,3-trimethylimidazolium cations. These results mean that the 2,6-methyl-N-methylpyridinium cation as an organic template might be more appropriate for directing the RTH zeolite structure [23]. In the following experiments, they found that the activation energy of RTH zeolite directed by this novel organic template was much lower than that of the conventional one. As a result, compared with the conventional synthesis (72 h at 130°C), the crystallization of RTH zeolite in this work took a very short time (50 min at 240°C and 12 h at 130°C). In addition, RTH zeolite exhibited high selectivity for propylene and ethylene of >75% in the MTO reaction, which would be of great importance for the selective production of light olefins in practical applications. Moreover, the Cu^2+^-exchanged RTH zeolite showed comparable performance to that of the commercial Cu-SSZ-13 zeolite, suggesting a promising zeolite for industrial applications in the NH_3_–SCR reaction.

SSZ-39 zeolite with an AEI structure is also a very important candidate for the NH_3_–SCR reaction [24–26]. However, Si/Al ratios in the conventional synthesis of AEI zeolite were <10, giving relatively low stability [24]. To enhance the stability, Schmidt *et al.* simulated the interaction energies between the zeolite framework with organic templates, predicting that a bulky organic template of N-ethyl-N-methyl-2,2,6,6-tetramethylpiperidinium has larger (more negative) stabilization energy than that of conventional organic templates for the formation of a high-silica SSZ-39 zeolite. Accordingly, they have successfully used this template for the synthesis of SSZ-39 with a Si/Al ratio of 13. As expected, it significantly enhanced the stability of the SSZ-39 zeolite, thus facilitating its application [27]. Considering the complexity and high cost of alkyl-substituted piperidinium cations in zeolite synthesis, Schwalbe-Koda *et al.* performed theoretical simulation by phase competition and shape analysis, reporting that a facile tris(dimethylamino)(methyl)phosphonium might be a novel candidate for synthesizing SSZ-39, which was confirmed experimentally. The obtained zeolite products displayed similar crystallinity and textural properties to those prepared with the conventional organic template, but the crystallization conditions are much milder [28].

In addition, Corma *et al.* showed a new method for the synthesis of zeolites by the employment of organic templates that mimic the transition state of pre-established reactions such as toluene disproportionation, ethylbenzene isomerization and adamantane synthesis [29]. Theoretical calculation displayed that the above organic templates are very suitable for synthesizing zeolites (ITQ-27, ITQ-64 and MIT-1). Later, they reported that small-pore zeolites with eight-membered rings (AEI, RTH and CHA) could also be successfully synthesized by using organic templates that mimic the key molecular species in the process of the MTO reaction [30].

### Preparation of zeolites with new composition

Although >250 zeolite frameworks have been discovered so far, there are only ∼20 zeolites being applied in industrial processes [31–33]. Among them, most of the zeolites are aluminosilicate compositions, which have both suitable acidic sites and excellent thermal and hydrothermal stabilities. Aluminogermanosilicate zeolites such as IWR and ITH, one of typical zeolite families, also exhibit excellent performance in the fields of sorption and catalysis [34–38], but the existence of germanium species in zeolite frameworks not only increase the zeolite cost, but also reduces their stabilities, which strongly limits their practical application [39,40]. Therefore, it is highly desirable to directly synthesize the aluminosilicate zeolites with the same structures as aluminogermanosilicate zeolites.

**Figure 3.**
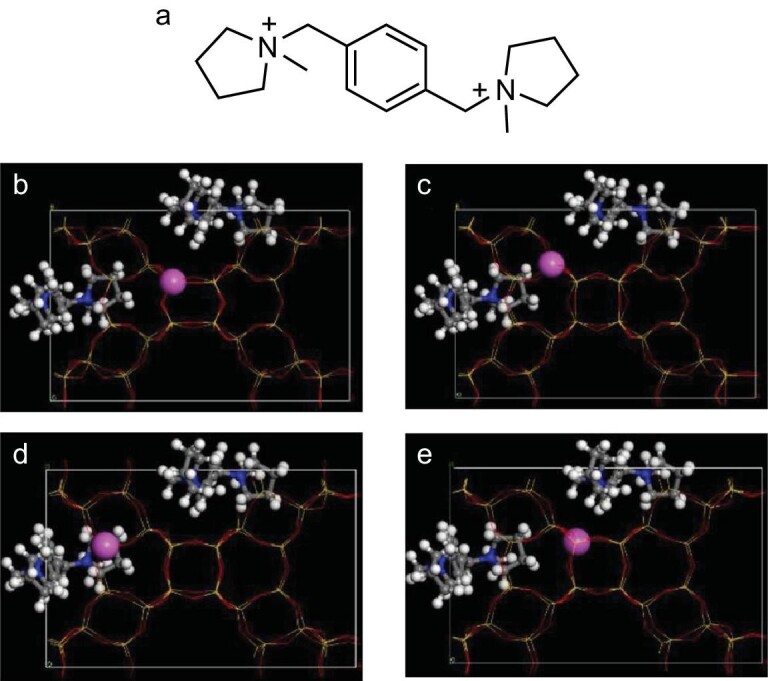
(a) The structure of an organic template and (b-e) the corresponding positions of organic templates in the aluminosilicate IWR zeolite structure with different T sites of Al species. Adapted with permission from ref. [41].

Hong *et al.* reported that aluminosilicate IWR zeolite could be directly synthesized using a designed organic template from a computational simulation screening by a strong interaction between a zeolite framework and pyrrolidine-based cations [41]. Based on minimum energy principle, it is suggested that larger stabilization energies (more negative) mean higher suitability for the zeolite synthesis. Balancing the interaction stabilization energy and cost of organic templates, a suitable organic template has been selected for directing the synthesis of targeted IWR zeolite, as shown in Fig. 3. As expected, the aluminosilicate IWR zeolite with a four-coordinated Al species (Al-IWR) has been successfully prepared from the designed organic template, in good agreement with the theoretical simulation. The Al-IWR zeolite could give an Si/Al ratio as low as 15. In methanol-to-propylene tests, the Al-IWR zeolite showed higher selectivity for propylene than that of commercial ZSM-5 zeolite, which might be of importance for the selective production of propylene from methanol—a platform compound from coal and biomass.

Later, Lei *et al.* reported the direct synthesis of aluminosilicate ITH zeolite by employing a designed cationic oligomer as an efficient organic template [42]. First, the theoretical simulation was performed to search for the suitable cationic oligomers. As observed from the interaction stabilization energy between the ITH framework and the structure of the cationic oligomer, it was suggested that the cationic oligomer was suitable for directing the crystallization of ITH zeolite. Second, it was found that the cationic oligomer had stronger complexation ability with aluminum species than that of the conventional organic template. Combining both advantages, aluminosilicate ITH zeolite was successfully synthesized. Moreover, aluminosilicate ITH zeolite exhibited excellent selectivity for propylene and a long lifetime in the MTP reaction. In addition, aluminosilicate ITH zeolite was a good additive for enhancing selectivities for butylene and propylene in the fluid catalytic cracking process. The employment of a cationic oligomer as an efficient organic template guided by theoretical simulation might open the door to directly preparing other aluminosilicate zeolites that could not be synthesized up to now.

### Development of novel routes for zeolite synthesis

The modern synthesis of zeolites normally requires the presence of organic templates and sometimes costly raw materials are necessary, which are environmentally unfriendly and have low efficiency [43]. Thus, developing novel routes for zeolite synthesis in a sustainable manner is highly desirable. Despite great efforts having been made in past decades for synthesizing zeolites [44–49], there are still many challenges in the field of zeolites synthesis with environmentally unfriendly features. For example, pure silica zeolites cannot be synthesized in the absence of organic templates and the use of organic templates both increases the zeolite cost and produces harmful gases. The synthesis of SSZ-39 zeolite is always performed in the presence of high-silica Y zeolite as a raw material and the use of this kind of high-silica Y zeolite is costly. To address these issues, both theoretical simulations and experimental work have been performed, and the typical examples are the organotemplate-free synthesis of pure silica MFI zeolite and the transformation of MFI and Beta zeolites into SSZ-39 zeolite.

**Figure 4.**
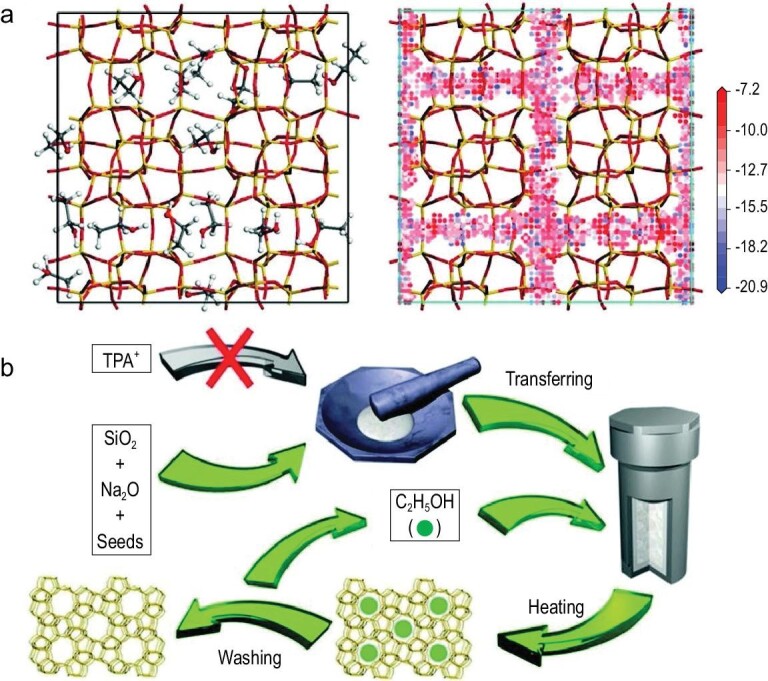
(a) The position of ethanol adsorbed in the micropore of silicalite-1 zeolite and the corresponding potential energy of isodensity surface for ethanol in silicalite-1 zeolite (blue represents low potential energy (kcal·mol^–1^)); (b) schematic representation for the synthesis of pure silica zeolites. Adapted with permission from ref. [53].

It is well known that pure silica zeolites have been widely used as catalytic supports and adsorbents in industrial processes [50–52], but their synthesis must use organic templates. Wu *et al.* for the first time showed that pure silica zeolites could be successfully synthesized in the presence of zeolite seeds and ethanol but in the absence of organic templates [53]. In this case, the zeolite seeds were used for directing zeolite growth, while the ethanol was employed for filling the zeolite pores. The filling role of ethanol is well confirmed by theoretical simulation, as shown in Fig. 4a. In the process of calculation, 16 ethanol molecules were fitted per unit cell in a silicalite-1 zeolite framework, giving an interaction energy of approximately –388.1 kcal/mol. On the contrary, only four TPABr molecules were fitted per unit cell in a silicalite-1 zeolite framework, giving an interaction energy of approximately –405.2 kcal/mol. The above results suggest that the ethanol molecules might just play the role of filling rather than directing. As expected, the sustainable synthesis of pure silica zeolites (MFI, MTT, TON and ^*^MRE) could be realized in the presence of zeolite seeds with ethanol as the directing and filling agent, respectively, as shown in Fig. 4b. In these examples, the calcination of pure silica zeolites obtained in this strategy is not necessary because the ethanol could be washed out from the zeolite micropore simply at room temperature. The filling role of ethanol in the zeolite micropore is evidenced by various characterization techniques such as XRD, TG, ^13^C MAS NMR, ^1^H MAS NMR and 2D ^1^H DQ-SQ MAS NMR.

**Figure 5.**
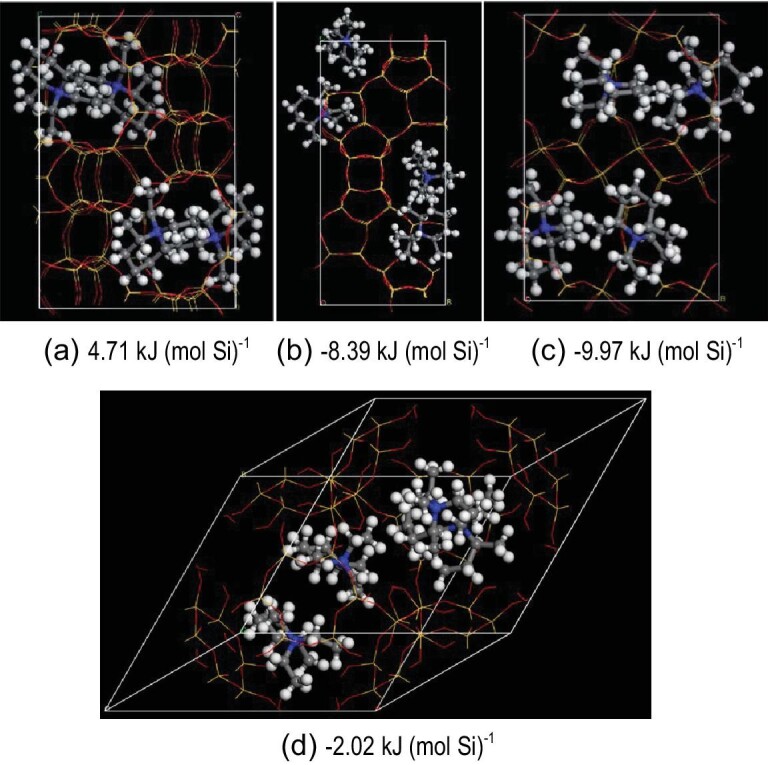
The positions of organic templates in the (a) ZSM-5, (b) Beta, (c) SSZ-39 and (d) Y zeolite structures and the corresponding stabilization energies between zeolite structures and organic templates. Adapted with permission from ref. [54].

SSZ-39 zeolite with an AEI structure is one of the most promising candidates instead of SSZ-13 zeolite used for the selective catalytic reduction of NO_*x*_ with NH_3_ (NH_3_–SCR). In general, SSZ-39 zeolite is synthesized by interzeolite transformation from high-silica Y zeolite, which is very costly [24]. Therefore, it is hopeful to use low-cost zeolites such as ZSM-5 and Beta as raw materials for the synthesis of SSZ-39 zeolite. However, it seems difficult because the interzeolite transformation from ZSM-5 and Beta zeolites into SSZ-39 zeolite would challenge the rule from a low framework density into a high framework density of zeolites. Notably, in published work, organic templates are always present for the interzeolite transformation but the contribution of organic templates for this transformation is completely ignored. If the interaction between organic templates with zeolite frameworks is considered, it is simulated that the stabilization energies between organic templates and zeolite structures (FAU, ^*^BEA and MFI) are higher than that between organic templates and AEI zeolite structures, which suggests that it is possible to synthesize SSZ-39 zeolite by interzeolite transformations from ZSM-5 and Beta zeolite, as shown in Fig. 5 [54]. Later experiments showed that the interzeolite transformation of SSZ-39 zeolite from ZSM-5 and Beta zeolites was successful. After being exchanged with Cu^2+^, the SSZ-39 zeolite exhibited extraordinary performance in the NH_3_–SCR reaction, which was fully comparable with that of SSZ-39 zeolite exchanged with Cu^2+^ from the conventional transformation synthesis.

Because raw materials such as colloidal silica are much cheaper than MFI and Beta zeolites, it is reasonable to directly synthesize SSZ-39 zeolite from colloidal silica. Initially, the as-synthesized products always form MOR zeolite rather than SSZ-39 zeolite. After the theoretical simulation of stabilization energies between organic templates and the zeolite structures (MOR and AEI), it is found that both stabilization energies are very low and similar, indicating that both zeolites could be formed for the synthesis of SSZ-39 zeolite. By adjusting the ratios of Si/Al in the starting materials for the avoidance of the MOR phase, it is successful for the direct synthesis of SSZ-39 zeolite using colloidal silica as a source [55], which further reduces the cost of SSZ-39 zeolite for practical applications.

### Synthesizing intergrowth zeolites

Compared with a sole-zeolite structure, intergrowth zeolites such as CHA/AEI, MFI/MEL, FAU/EMT, MTT/TON and SBS/SBT always give distinctive properties leading to excellent performance for practical applications [56–60]. In general, the synthesis of intergrowth zeolites was performed in the presence of dual organic templates, which required fine adjustment of the synthetic parameters to avoid the formation of separated zeolite phases. Thus, it is expected to design a bi-selective organic template for directing the targeted intergrowth zeolites.

**Figure 6.**
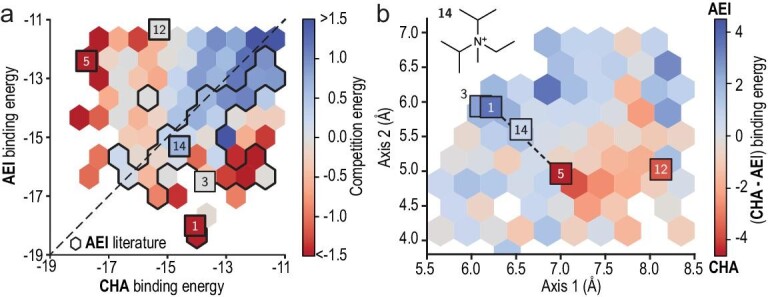
(a) The comparison between the binding energies of organic templates in AEI and CHA zeolites; (b) the relationship between the shapes of organic templates and the corresponding binding energies of AEI and CHA zeolites. Adapted with permission from ref. [28].

Schwalbe-Koda *et al.* for the first time reported the formation of aluminosilicate CHA/AEI intergrowth zeolite from N-ethyl-N-isopropyl-N-methylpropan-2-aminium as an organic template through theoretical calculations. This template has similar binding energies to both pure CHA and AEI phases as well as its shape positioned on the phase boundary between the CHA and AEI structures, as shown in Fig. 6. Based on theoretical calculation, the aluminosilicate CHA/AEI zeolite was successfully synthesized under suitable conditions. The intergrowth property of CHA/AEI zeolite was confirmed by the SEM, XRD and HRTEM techniques. In addition, the result of DIFFaX simulation confirmed the CHA/AEI intergrowth ratio at ∼1.0 [28]. Later, they employed computational simulations and data mining to design bi-selective organic templates used for directing formation of other intergrown zeolites (BEC/ISV, MTT/TON and CHA/AFX). Furthermore, a bi-selective organic template for the potential formation of a hypothesized intergrowth zeolite structure (AEI/SAV) was also proposed [61].

### Generation of novel zeolite structures

The generation of novel zeolite structures is always a hot topic in the field of zeolite research [62–70]. Currently, most novel zeolite structures are made from organic templates via a trial-and-error route, which is time-consuming and has low efficiency. One efficient method is to use theoretical simulation.

**Figure 7.**
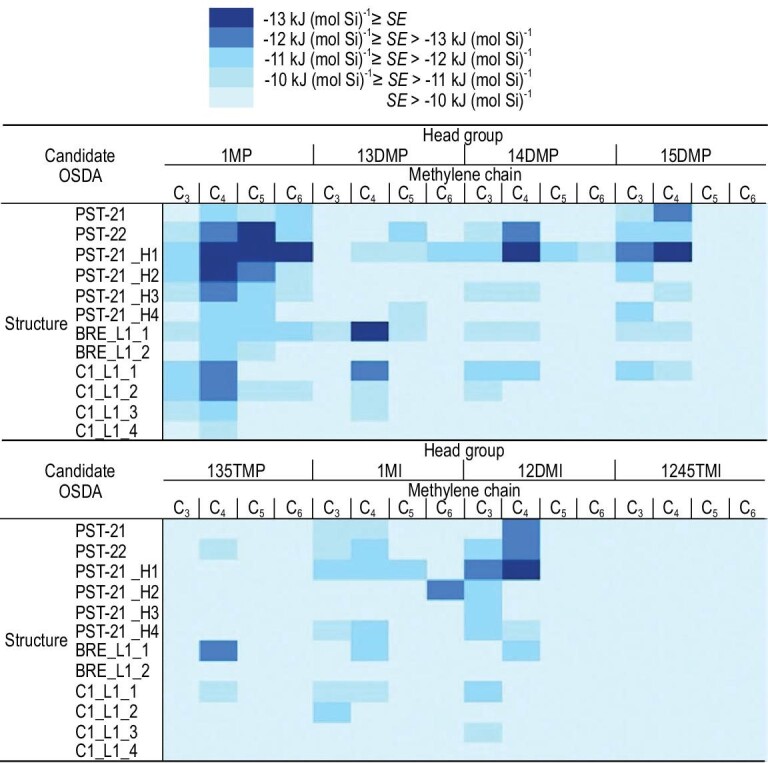
The stabilization energies of the 32 organic templates in PST-22, PST-21 and hypothetical zeolite structures. Each organic template is made up of methyl-substituted diazole linked a chain of methylene with three to six carbon atoms. Adapted with permission from ref. [71].

A typical example reported by Hong *et al.* is the successful synthesis of a novel zeolite structure of PST-30 guided by calculating the stabilization energies between organic templates and hypothetical zeolite structures, as shown in Fig. 7 [71]. At first, the stabilization energies of the reported organic templates for PST-21 and PST-22 zeolites were calculated [72], showing that 1,2,3-trimethylimidazolium had a stabilization energy of –13.7 kJ (mol Si)^–1^ in the PST-21 zeolite structure, while 1,3,4-trimethylimidazolium and 1,2,3,4-tetramethylimidazolium gave the stabilization energies of approximately –13.4 and –13.8 kJ (mol Si)^–1^ in the PST-22 zeolite structure. Therefore, the benchmark energy for considering whether organic templates would direct the formation of novel targeted zeolite structures was set at –13.0 kJ (mol Si)^–1^. After calculating the stabilization energies by interaction between pyrazolium-based and diazole-based organic templates with hypothetical zeolite structures, it was found that [1,1^′^-(1,4-butane-diyl)bis(2,5-dimethyl-1H-pyrazol-2-ium)] might be a suitable candidate for directing BRE_L1_1 (PST-30) zeolite due to its large stabilization energy (more negative) by computational simulation. After the introduction of this template into the synthesis system, PST-30 zeolite with good crystallinity could be successfully synthesized under suitable crystallization conditions, as confirmed by the XRD technique, in good agreement with the simulated XRD pattern. The structure analysis of PST-30 zeolite showed that it had a 2D micropore consisting of 8-MRs (4.2 × 4.4 Å) and 10-MRs (4.7 × 6.7 Å). This special micropore system of PST-30 zeolite displayed excellent catalytic performance in the skeletal isomerization of the 1-butene to isobutene reaction. The targeted synthesis of the novel zeolite structure chosen from the database of hypothetical zeolites might open the door for synthesizing other novel zeolite structures guided by computational calculations of organic templates.

### Control of zeolite morphology

Selective control of zeolite morphologies such as nanosheets, nanoparticles and nanoneedles is of great importance due to fast mass transfer [73–82]. Recently, great efforts have been witnessed for this kind of work [83,84] and one line of progress has been to control zeolite morphology assisted with theoretical simulation.

**Figure 8.**
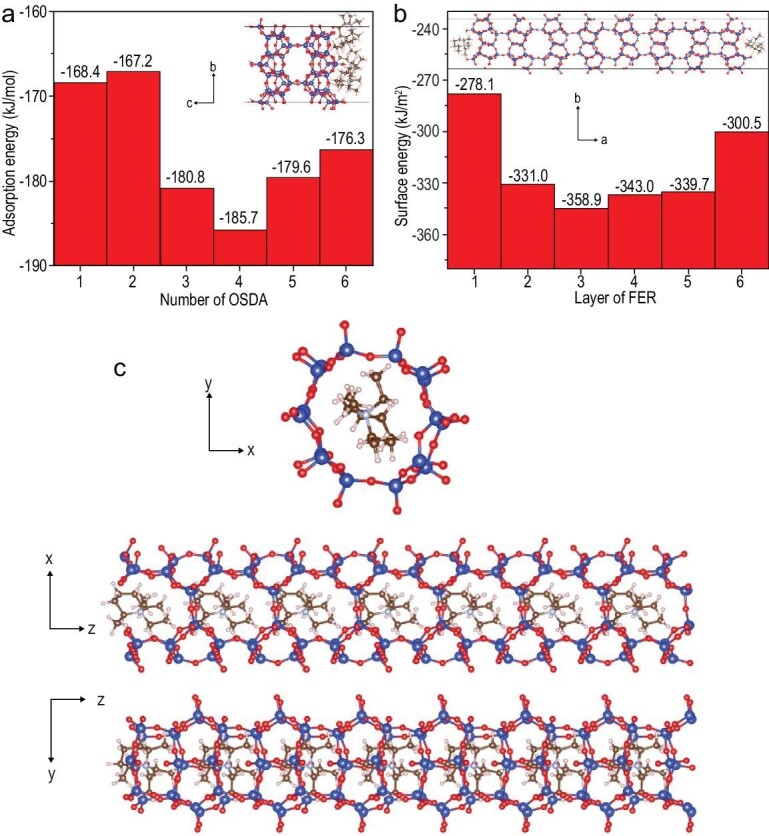
(a) Dependence of adsorption energy on the surfaces of FER zeolite on the number of organic template molecules and the corresponding schematic diagram; (b) dependence of the surface energy of FER zeolite on layers of its structure and the corresponding schematic diagram; (c) position of organic template molecules in the FER structure framework. Adapted with permission from ref. [85].

Xu *et al.* reported that ultra-thin nanosheets of FER zeolite (N-FER) with a thickness of ∼8 nm were successfully synthesized by the employment of a small organic ammonium salt (N,N-diethyl-*cis*-2,6-dimethylpiperidinium, DMP) [85]. Theoretical simulations were used for understanding the N-FER zeolite with four-unit-cells along with the [100] direction. Figure 8a shows the calculated adsorption energy associated with the number of DMP molecules on FER [100] surfaces, showing that the model with four DMP molecules had the largest adsorption energy (the most negative). Then, the dependence of the surface energy of FER zeolite on layers of its structure was calculated, showing that three- to five-layered zeolite structure is stable, as shown in Fig. 8b. The above results indicated that the DMP molecules would adsorb on the [100] plane of FER zeolite, which limited the FER layers’ growth and thus formed the ultra-thin nanosheets FER zeolite with a thickness of ∼8 nm. Furthermore, theoretical calculation also predicted the position of the DMP molecules in the FER zeolite structure, showing that two DMP molecules per unit cell had the largest stabilization energy (the most negative), as shown in Fig. 8c. This result suggests that the DMP is an efficient organic template for directing the formation of a FER zeolite structure. In addition, the N-FER zeolite exhibited higher conversion and isomer selectivity than the conventional FER zeolite due to the feature of ultra-thin nanosheets.

Later, Peng *et al.* showed that a small, cheap and simple imidazole-based molecule could not only direct the formation of FER zeolite structure, but also limit the growth of the FER zeolite nanosheet by π–π stacking interaction [86]. As a result, ultra-thin FER zeolite nanosheets (ECNU-17) with a thickness of only 2.9 nm could be successfully synthesized. Theoretical simulation showed that the ECNU-17 nanosheets formed easily when two layers of organic template molecules were positioned on the external surface of the layers. Due to a significant increase in accessible acidic sites, the ultra-thin FER zeolite exhibited superior catalytic performance in the catalytic cracking of 1,3,5-triisopropylbenzene, a model compound for bulky molecules.

### Modulation of aluminum distribution in zeolites

Aluminosilicate zeolites as acidic catalysts have been widely applied for petrochemical processes. Notably, the same aluminosilicate zeolites sometimes show quite different performances, which are related to the distinguished aluminum distribution governed by the different synthetic parameters [87–94]. Among these parameters, the role of organic templates is critical for the modulation of aluminum distribution in zeolites, where the development of computational simulation techniques offers a good chance for the design of organic templates.

**Figure 9.**
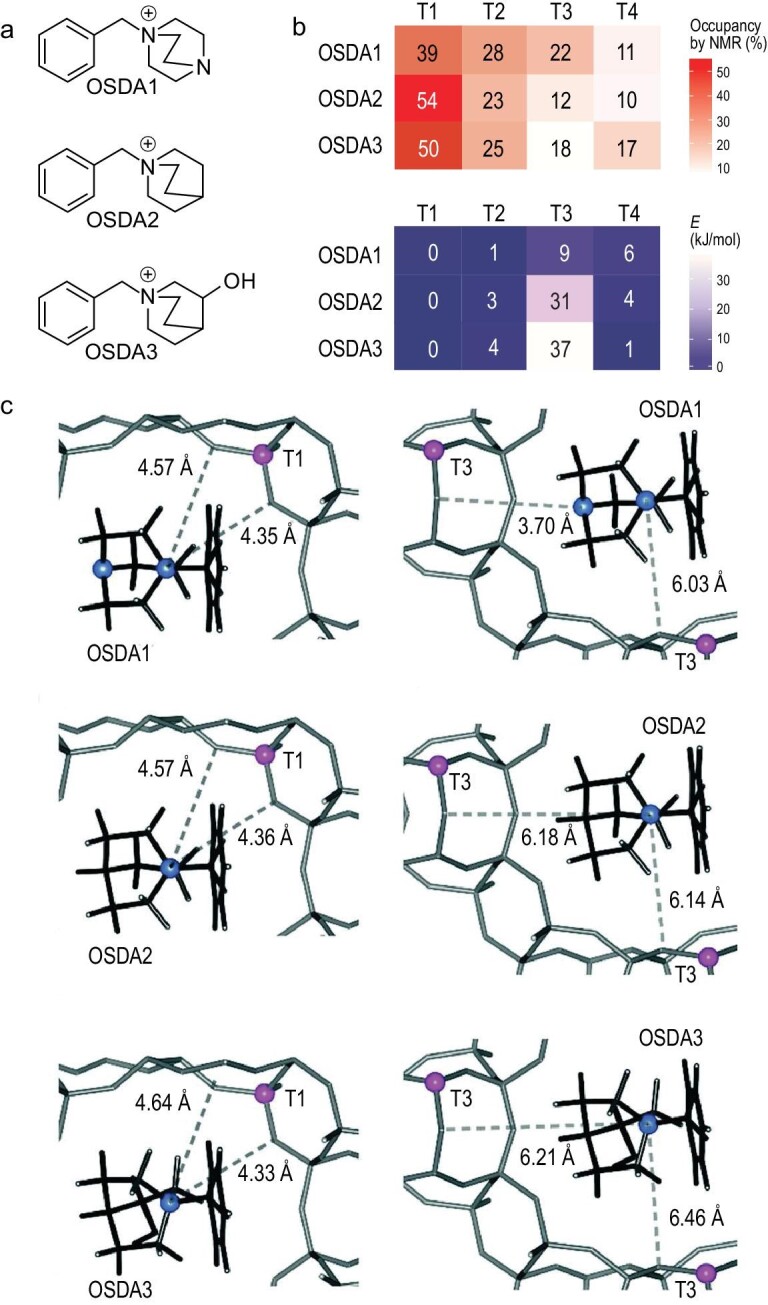
(a) The chemical structures of three organic templates; (b) heat map of aluminum positions and their relative energies of IFR zeolite prepared in the presence of the above organic templates; (c) the optimized structures of zeolite and organic template complexes (the blue spheres represent N, and the pink spheres represent Al). Adapted with permission from ref. [95].

Muraoka *et al.* reported the preparation of IFR zeolites with controllable aluminum distribution at different T sites using three different organic templates. Theoretical simulation showed that the T1 site was the most stable position for aluminum for any of the organic templates, which was in good agreement with the result from the MAS NMR technique. At the same time, short N^+^...O(Al) distances would be observed for three organic templates and the T1 site, which confirms the highest occupancy of the T1 site in three products. On the contrary, theoretical simulation showed the highest energy at the T3 site confirmed by the long N^+^...O(Al) distances, which led to the low occupancy in the T3 site of products, as shown in Fig. 9 [95].

Chu *et al.* demonstrated the constrained aluminum sites in aluminosilicate FER zeolite. Theoretical simulation was performed by interaction between the FER zeolite structure and protonated pyridines. The results showed that the T1 and T3 sites of FER zeolite were the preferable occupancy, in good agreement with the results of Rietveld refinement. As a result, the above FER zeolite displayed limited catalytic performance in the carbonylation of dimethyl ether since the acidic sites of T1 and T3 in the open channels cannot act as the active center for this reaction [96].

More recently, Schwalbe-Koda *et al.* showed that the aluminum distribution of SSZ-13 zeolite was associated with the spatial charge distribution of organic templates through theoretical simulation. The nitrogen center descriptor showed that the specific organic template with the N center was close to the center of the CHA cage, which could make it difficult to form more paired aluminum sites [28]. The above principle might be a good pathway to the modulation of aluminum distribution in SSZ-13 zeolite, then improving the performance of SSZ-13 zeolite in the NH_3_–SCR reaction.

## CONCLUSION AND PERSPECTIVE

In summary, the targeted synthesis of zeolites from the calculated interaction between zeolite structures and organic templates has been briefly concluded. The targeted syntheses of zeolites mainly include the design of new templates for zeolite synthesis, preparation of zeolites with new composition, development of novel routes for zeolite synthesis, synthesis of intergrowth zeolites, generation of novel zeolite structures, control of zeolite morphology and modulation of aluminum distribution in the zeolites. In these examples, it is revealed that the minimum energy principle for the calculated interaction energies between zeolite frameworks and organic templates is critical for the successful experimental synthesis of targeted zeolites. Of course, a minimum energy calculation is capturing the local minimum in the potential energy surface. Following this way, it is believable that more work is still on the way for the targeted synthesis of zeolites guided by theoretical simulation.

Despite great efforts having been made for the targeted synthesis of zeolites, there are still challenges to be addressed. For example, many zeolite structures show excellent catalytic performances but their syntheses are costly due to the use of complex organic templates; many zeolite structures are easily synthesized in the lab but they are difficult for scale-up preparation for industrial production. Possibly, theoretical simulation offers an alternative way to solve these kinds of problems in the future.

**Figure 10.**
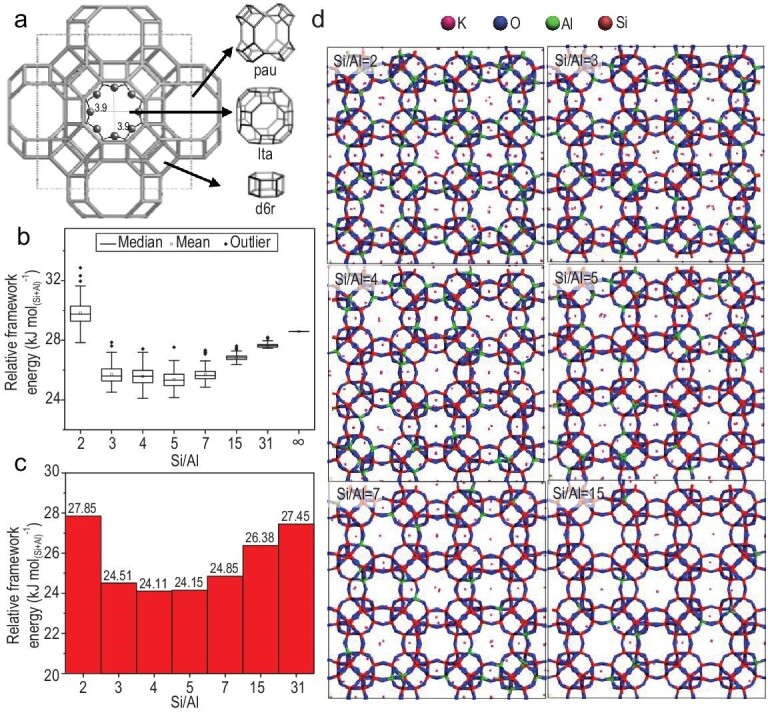
(a) KFI zeolite structure along [100] and its building units; (b) statistical plot of the relative framework energy versus Si/Al ratios for K-KFI zeolite; (c) the minimum relative framework energy of KFI zeolites with each Si/Al ratio; (d) the most stable KFI zeolite structures for each Si/Al ratio. Adapted with permission from ref. [97].

Considering the costly organic templates in many zeolite syntheses, it is strongly desirable to use inorganic templates such K^+^ cation instead of organic templates for synthesizing zeolites, which can be guided from the calculated interaction energies between zeolite frameworks and inorganic cations. For example, Han *et al.* showed the successful preparation of a new high-silica KFI zeolite with a Si/Al ratio of >5.0 in the presence of K^+^ cation but in the absence of any organic templates, which is effectively inspired by interaction energies between the zeolite structure and the K^+^ cation, as shown in Fig. 10 [97]. The simulated interaction energies between zeolite frameworks and inorganic cations might help to experimentally synthesize more zeolite structures in the future.

However, the targeted synthesis of zeolite guided by computational simulation also has technical challenges and limitation. For example, the effect of synthetic conditions such as having water as a solvent are not often considered in computational simulations; the defects in targeted zeolite frameworks are usually ignored in theoretical calculations. In addition, it is worth noting that the zeolites are metastable in terms of thermodynamics, which are often influenced by many synthetic parameters [98,99].

Possibly, the targeted synthesis of zeolites guided by theoretical simulation would open a new door for synthesizing zeolites, which is should be efficient, low-cost and time-saving. Therefore, more efforts should be made for this kind of research in the future.

## References

[1] International Zeolite Association Structure Commission . Database of Zeolite Structures. https://asia.iza-structure.org/IZA-SC/ftc_table.php (16 July 2021, date last accessed).

[2] Xie Z , Liu Z, Wang Y et al. Applied catalysis for sustainable development of chemical industry in China. Natl Sci Rev 2015; 2: 167–82. 10.1093/nsr/nwv019

[3] Sun Q , Xie Z, Yu J et al. The state-of-the-art synthetic strategies for SAPO-34 zeolite catalysts in methanol-to-olefin conversion. Natl Sci Rev 2018; 5: 542–58. 10.1093/nsr/nwx103

[4] Chen L , Sun M, Wang Z et al. Hierarchically structured zeolites: from design to application. Chem Rev 2020; 120: 11194–294. 10.1021/acs.chemrev.0c00016 32915551

[5] Mintova S , Gilson J, Valtchev V et al. Advances in nanosized zeolites. Nanoscale 2013; 5: 6693–703. 10.1039/c3nr01629c 23803972

[6] Schwieger W , Machoke A, Weissenberger T et al. Hierarchy concepts: classification and preparation strategies for zeolite containing materials with hierarchical porosity. Chem Soc Rev 2016; 45: 3353–76. 10.1039/C5CS00599J 26477329

[7] Zhang Q , Yu J, Corma A. Applications of zeolites to C1 chemistry: recent advances, challenges, and opportunities. Adv Mater 2020; 32: 2002927. 10.1002/adma.202002927 32697378

[8] Chai Y , Dai W, Wu G et al. Confinement in a zeolite and zeolite catalysis. Acc Chem Res 2021; 54: 2894–904. 10.1021/acs.accounts.1c00274 34165959

[9] Dusselier M , Davis M. Small-pore zeolites: synthesis and catalysis. Chem Rev 2018; 118: 5265–329. 10.1021/acs.chemrev.7b00738 29746122

[10] Peng P , Gao X, Yan Z et al. Diffusion and catalyst efficiency in hierarchical zeolite catalysts. Natl Sci Rev 2020; 7: 1726–42. 10.1093/nsr/nwaa184 34691504 PMC8290962

[11] Galli E , Quartieri S, Vezzalini G et al. Boggsite and Tschernichite-type zeolites from Mt. Adamson, Northern Victoria Land (Antarctica). Eur J Mineral 1995; 7: 1029–32. 10.1127/ejm/7/4/1029

[12] Simancas R , Dari D, Velamazan N et al. Modular organic structure-directing agents for the synthesis of zeolites. Science 2010; 330: 1219–22. 10.1126/science.1196240 21109667

[13] Rojas A , Camblor M. A pure silica chiral polymorph with helical pores. Angew Chem Int Ed 2012; 51: 3854–6. 10.1002/anie.201108753 22392818

[14] Rojas A , Arteaga O, Kahr B et al. Synthesis, structure, and optical activity of HPM-1, a pure silica chiral zeolite. J Am Chem Soc 2013; 135: 11975–84. 10.1021/ja405088c 23865767

[15] Schmidt J , Deem M, Davis M et al. Synthesis of a specified, silica molecular sieve by using computationally predicted organic structure-directing agents. Angew Chem Int Ed 2014; 53: 8372–4. 10.1002/anie.201404076 24961789

[16] Xie D , McCusker L, Baerlocher C et al. SSZ-52, a zeolite with an 18-layer aluminosilicate framework structure related to that of the DeNO_x_ catalyst Cu-SSZ-13. J Am Chem Soc 2013; 135: 10519–24. 10.1021/ja4043615 23782259

[17] Li R , Zhu Y, Zhang Z et al. Remarkable performance of selective catalytic reduction of NO_x_ by ammonia over copper-exchanged SSZ-52 catalysts. Appl Catal B 2021; 283: 119641. 10.1016/j.apcatb.2020.119641

[18] Davis T , Liu A, Lew C et al. Computationally guided synthesis of SSZ-52: a zeolite for engine exhaust clean-up. Chem Mater 2016; 28: 708–11. 10.1021/acs.chemmater.5b04578

[19] Yokoi T , Yoshioka M, Ima H et al. Diversification of RTH-type zeolite and its catalytic application. Angew Chem Int Ed 2009; 48: 9884–7. 10.1002/anie.200905214 19937889

[20] Schmidt J , Deimund M, Davis M et al. Facile preparation of aluminosilicate RTH across a wide composition range using a new organic structure-directing agent. Chem Mater 2014; 26: 7099–105. 10.1021/cm503625u

[21] Shan Y , Shi X, Du J et al. Cu-exchanged RTH-type zeolites for NH_3_-selective catalytic reduction of NO_x_: Cu distribution and hydrothermal stability. Catal Sci Technol 2019; 9: 106–15. 10.1039/C8CY01933A

[22] Jo D , Lim J, Ryu T et al. Unseeded hydroxide-mediated synthesis and CO_2_ adsorption properties of an aluminosilicate zeolite with the RTH topology. J Mater Chem A 2015; 3: 19322–9. 10.1039/C5TA03559G

[23] Xu H , Chu Y, Wu Q et al. Efficient synthesis of aluminosilicate RTH zeolite with good catalytic performances in NH_3_-SCR and MTO reactions. J Mater Chem A 2018; 6: 8705–11. 10.1039/C8TA01734D

[24] Moliner M , Franch C, Palomares E et al. Cu-SSZ-39, an active and hydrothermally stable catalyst for the selective catalytic reduction of NO_x_. Chem Commun 2012; 48: 8264–6. 10.1039/c2cc33992g 22782014

[25] Du J , Shan Y, Sun Y et al. Unexpected increase in low-temperature NH_3_-SCR catalytic activity over Cu-SSZ-39 after hydrothermal aging. Appl Catal B 2021; 294: 120237. 10.1016/j.apcatb.2021.120237

[26] Zhu N , Shan Y, Shan W et al. Distinct NO_2_ effects on Cu-SSZ-13 and Cu-SSZ-39 in the selective catalytic reduction of NO_x_ with NH_3_. Environ Sci Technol 2020; 54: 15499–506. 10.1021/acs.est.0c06256 33200925

[27] Schmidt J , Deem M, Lew C et al. Computationally-guided synthesis of the 8-ring zeolite AEI. Top Catal 2015; 58: 410–5. 10.1007/s11244-015-0381-1

[28] Schwalbe-Koda D , Kwon S, Paris C et al. A priori control of zeolite phase competition and intergrowth with high-throughput simulations. Science 2021; 374: 308–15. 10.1126/science.abh3350 34529493

[29] Maria G , Teresa P, Paris C et al. ‘Ab initio’ synthesis of zeolites for preestablished catalytic reactions. Science 2017; 355: 1051–4. 28280200 10.1126/science.aal0121

[30] Li C , Paris C, Martinez-Triguero J et al. Synthesis of reaction-adapted zeolites as methanol-to-olefins catalysts with mimics of reaction intermediates as organic structure-directing agents. Nat Catal 2018; 1: 547–54. 10.1038/s41929-018-0104-7

[31] Cundy C , Cox P. The hydrothermal synthesis of zeolites: history and development from the earliest days to the present time. Chem Rev 2003; 103: 663–702. 10.1021/cr020060i 12630849

[32] Li Y , Li L, Yu J et al. Applications of zeolites in sustainable chemistry. Chem 2017; 3: 928–49. 10.1016/j.chempr.2017.10.009

[33] Tian P , Wei Y, Ye M et al. Methanol to olefins (MTO): from fundamentals to commercialization. ACS Catal 2015; 5: 1922–38. 10.1021/acscatal.5b00007

[34] Li L , Chen Y, Xu S et al. Oriented control of Al locations in the framework of Al-Ge-ITQ-13 for catalyzing methanol conversion to propene. J Catal 2016; 344: 242–51. 10.1016/j.jcat.2016.09.007

[35] Xie Z , Liu Z, Wang Y et al. Rational design and HT techniques allow the synthesis of new IWR zeolite polymorphs. J Am Chem Soc 2006; 128: 4216–7. 16568982 10.1021/ja0603599

[36] Liu X , Mao W, Jiang J et al. Topotactic conversion of alkali-treated intergrown germanosilicate CIT-13 into single-crystalline ECNU-21 zeolite as shape-selective catalyst for ethylene oxide hydration. Chem Eur J 2019; 25: 4520–9. 10.1002/chem.201900173 30698847

[37] Kasian N , Verheyen E, Vanbutsele G et al. Catalytic activity of germanosilicate UTL zeolite in bifunctional hydroisomerisation of n-decane. Microporous Mesoporous Mater 2013; 166: 153–60. 10.1016/j.micromeso.2012.07.017

[38] Fu W , Yuan Z, Wang Z et al. Direct synthesis of hydrothermally stable Ge-IWR zeolites. Dalton Trans 2017; 46: 6692–9. 10.1039/C7DT01087G 28484777

[39] Shvets O , Kasian N, Zukal A et al. The role of template structure and synergism between inorganic and organic structure directing agents in the synthesis of UTL zeolite. Chem Mater 2010; 22: 3482–95. 10.1021/cm1006108

[40] Shamzhy M , Shvets O, Opanasenko M et al. Synthesis of isomorphously substituted extra-large pore UTL zeolites. J Mater Chem 2012; 22: 15793–803. 10.1039/c2jm31725g

[41] Hong X , Chen W, Zhang G et al. Direct synthesis of aluminosilicate IWR zeolite from a strong interaction between zeolite framework and organic template. J Am Chem Soc 2019; 141: 18318–24. 10.1021/jacs.9b09903 31644275

[42] Lei C , Dong Z, Martinez C et al. A cationic oligomer as an organic template for direct synthesis of aluminosilicate ITH zeolite. Angew Chem Int Ed 2020; 59: 15649–55. 10.1002/anie.202003282 32453899

[43] Cejka J , Van Bekkum H, Corma A et al. Introduction to Zeolite Molecular Sieves. Amsterdam: Elsevier Science, 2007.

[44] Wu Q , Meng X, Gao X et al. Solvent-free synthesis of zeolites: mechanism and utility. Acc Chem Res 2018; 51: 1396–403. 10.1021/acs.accounts.8b00057 29738233

[45] Liu Z , Zhu Z, Wakihara T et al. Ultrafast synthesis of zeolites: breakthrough, progress and perspective. Inorg Chem Front 2019; 6: 14–31. 10.1039/C8QI00939B

[46] Meng X , Xiao F. Green routes for synthesis of zeolites. Chem Rev 2014; 114: 1521–43. 10.1021/cr4001513 24187944

[47] Kasneryk V , Shamzhy M, Zhou J et al. Vapor-phase-transport rearrangement technique for the synthesis of new zeolites. Nat Commun 2019; 10: 5129. 10.1038/s41467-019-12882-3 31719520 PMC6851152

[48] Li R , Linares N, Sutjianto J et al. Ultrasmall zeolite L crystals prepared from highly interdispersed alkali-silicate precursors. Angew Chem Int Ed 2018; 57: 11283–8. 10.1002/anie.201805877 29920889

[49] Zhang X , Liu D, Xu D et al. Synthesis of self-pillared zeolite nanosheets by repetitive branching. Science 2012; 336: 1684–7. 10.1126/science.1221111 22745424

[50] Wang Z , Wang H, Mitra A et al. Pure-silica zeolite low-k dielectric thin films. Adv Mater 2001; 13: 746–9. 10.1002/1521-4095(200105)13:10<746::AID-ADMA746>3.0.CO;2-J

[51] Bereciartua P , Cantin A, Corma A et al. Control of zeolite framework flexibility and pore topology for separation of ethane and ethylene. Science 2017; 358: 1068–71. 10.1126/science.aao0092 29170235

[52] Vattipalli V , Paracha A, Hu W et al. Broadening the scope for fluoride-free synthesis of siliceous zeolites. Angew Chem Int Ed 2018; 57: 3607–11. 10.1002/anie.201712684 29377484

[53] Wu Q , Zhu L, Chu Y et al. Sustainable synthesis of pure silica zeolites from a combined strategy of zeolite seeding and alcohol filling. Angew Chem Int Ed 2019; 58: 12138–42. 10.1002/anie.201906559 31283076

[54] Xu H , Chen W, Wu Q et al. Transformation synthesis of aluminosilicate SSZ-39 zeolite from ZSM-5 and beta zeolite. J Mater Chem A 2019; 7: 4420–5. 10.1039/C9TA00174C

[55] Xu H , Zhang J, Wu Q et al. Direct synthesis of aluminosilicate SSZ-39 zeolite using colloidal silica as a starting source. ACS Appl Mater Interfaces 2019; 11: 23112–7. 10.1021/acsami.9b03048 31252486

[56] Sun Q , Ma Y, Wang N et al. High performance nanosheet-like silicoaluminophosphate molecular sieves: synthesis, 3D EDT structural analysis and MTO catalytic studies. J Mater Chem A 2014; 2: 17828–39. 10.1039/C4TA03419H

[57] Zhang L , Liu S, Xie S et al. Organic template-free synthesis of ZSM-5/ZSM-11 co-crystalline zeolite. Microporous Mesoporous Mater 2012; 147: 117–26. 10.1016/j.micromeso.2011.05.033

[58] Khaleel M , Wagner A, Mkhoyan K et al. On the rotational intergrowth of hierarchical FAU/EMT zeolites. Angew Chem Int Ed 2014; 53: 9456–61. 10.1002/anie.201402024 25044073

[59] Burton A , Zones S, Rea T et al. Preparation and characterization of SSZ-54: a family of MTT/TON intergrowth materials. Microporous Mesoporous Mater 2010; 132: 54–9. 10.1016/j.micromeso.2009.10.023

[60] Lee H , Shin J, Lee K et al. Synthesis of thermally stable SBT and SBS/SBT intergrowth zeolites. Science 2021; 373: 104–7. 10.1126/science.abi7208 34210885

[61] Schwalbe-Koda D , Corma A, Roman-Leshkov Y et al. Data-driven design of biselective templates for intergrowth zeolites. J Phys Chem Lett 2021; 12: 10689–94. 10.1021/acs.jpclett.1c03132 34709806

[62] Gao Z , Li J, Lin C et al. HPM-14: a new germanosilicate zeolite with interconnected extra-large pores plus odd-membered and small pores. Angew Chem Int Ed 2021; 60: 3438–42. 10.1002/anie.202011801 33140883

[63] Opanasenko M , Shamzhy M, Wang Y et al. Synthesis and post-synthesis transformation of germanosilicate zeolites. Angew Chem Int Ed 2020; 59: 19380–9. 10.1002/anie.202005776 32510709

[64] Trachta M , Bludsky O, Cejka J et al. From double-four-ring germanosilicates to new zeolites: in silico investigation. ChemPhysChem 2014; 15: 2972–6. 10.1002/cphc.201402358 25048804

[65] Smeets S , Zones S, Xie D et al. SSZ-27: a small-pore zeolite with large heart-shaped cavities determined by using multi-crystal electron diffraction. Angew Chem Int Ed 2019; 58: 13080–6. 10.1002/anie.201905049 PMC6773097 31347746

[66] Smeets S , Xie D, Baerlocher C et al. High-silica zeolite SSZ-61 with dumbbell-shaped extra-large-pore channels. Angew Chem Int Ed 2014; 53: 10398–402. 10.1002/anie.201405658 25088447

[67] Nakazawa N , Ikeda T, Hiyoshi N et al. A microporous aluminosilicate with 12-, 12-, and 8-ring pores and isolated 8-ring channels. J Am Chem Soc 2017; 139: 7989–97. 10.1021/jacs.7b03308 28581728

[68] Mazur M , Wheatley P, Navarro M et al. Synthesis of ‘unfeasible’ zeolites. Nat Chem 2016; 8: 58–62. 10.1038/nchem.2374 26673264

[69] Eliasova P , Opanasenko M, Wheatley P et al. The ADOR mechanism for the synthesis of new zeolites. Chem Soc Rev 2015; 44: 7177–206. 10.1039/C5CS00045A 25946705

[70] Shin J , Jo D, Hong S et al. Rediscovery of the importance of inorganic synthesis parameters in the search for new zeolites. Acc Chem Res 2019; 52: 1419–27. 10.1021/acs.accounts.9b00073 31013053

[71] Jo D , Hong S. Targeted synthesis of a zeolite with pre-established framework topology. Angew Chem Int Ed 2019; 58: 13845–8. 10.1002/anie.201909336 31359574

[72] Jo D , Park G, Shin J et al. A zeolite family nonjointly built from the 1,3-stellated cubic building unit. Angew Chem Int Ed 2018; 57: 2199–203. 10.1002/anie.201712885 29251386

[73] Margarit V , Diaz-Rey M, Navarro M et al. Direct synthesis of nano-ferrierite along the 10-ring-channel direction boosts their catalytic behavior. Angew Chem Int Ed 2018; 57: 3459–63. 10.1002/anie.201711418 29485242

[74] Lu K , Huang J, Ren L et al. High ethylene selectivity in methanol-to-olefin (MTO) reaction over MOR-zeolite nanosheets. Angew Chem Int Ed 2020; 59: 6258–62. 10.1002/anie.202000269 31981394

[75] Dai W , Kouvatas C, Tai W et al. Plate-like MFI crystals with controlled crystal faces aspect ratio. J Am Chem Soc 2021; 143: 1993–2004. 10.1021/jacs.0c11784 33464884

[76] Jain R , Chawla A, Linares N et al. Spontaneous pillaring of pentasil zeolites. Adv Mater 2021; 33: 2100897. 10.1002/adma.202100897 33904205

[77] Lee Y , Park M, Kim P et al. Synthesis and catalytic behavior of ferrierite zeolite nanoneedles. ACS Catal 2013; 3: 617–21. 10.1021/cs400025s

[78] Zhao X , Zeng S, Zhang X et al. Generating assembled MFI nanocrystals with reduced b-axis through structure-directing agent exchange induced recrystallization. Angew Chem Int Ed 2021; 60: 13959–68. 10.1002/anie.202017031 33844380

[79] Choi M , Na K, Kim J et al. Nanosheets of zeolite MFI as active and long-lived catalysts. Nature 2009; 461: 246–9. 10.1038/nature08288 19741706

[80] Xu D , Ma Y, Jing Z et al. π–π interaction of aromatic groups in amphiphilic molecules directing for single-crystalline mesostructured zeolite nanosheets. Nat Commun 2014; 5: 4262. 10.1038/ncomms5262 24957696

[81] Na K , Jo C, Kim J et al. Directing zeolite structures into hierarchically nanoporous architectures. Science 2011; 333: 328–32. 10.1126/science.1204452 21764745

[82] Chaikittisilp W , Suzuki Y, Mukti R et al. Formation of hierarchically organized zeolites by sequential intergrowth. Angew Chem Int Ed 2013; 52: 3355–9. 10.1002/anie.201209638 23418100

[83] Li S , Li J, Dong M et al. Strategies to control zeolite particle morphology. Chem Soc Rev 2019; 48: 885–907. 10.1039/C8CS00774H 30601526

[84] Roth W , Sasaki T, Wolski K et al. Exfoliated ferrierite-related unilamellar nanosheets in solution and their use for preparation of mixed zeolite hierarchical structures. J Am Chem Soc 2021; 143: 11052–62. 10.1021/jacs.1c04081 34264655 PMC8397323

[85] Xu H , Chen W, Zhang G et al. Ultrathin nanosheets of aluminosilicate FER zeolites synthesized in the presence of a sole small organic ammonium. J Mater Chem A 2019; 7: 16671–6. 10.1039/C9TA04833B

[86] Peng M , Wang Z, Huang J et al. Two coexisting forms of simple molecules for directing sesqui-unit-cell zeolite nanosheets. Chem Mater 2021; 33: 6934–41. 10.1021/acs.chemmater.1c01876

[87] Yokoi T , Mochizuki H, Namba S et al. Control of the Al distribution in the framework of ZSM-5 zeolite and its evaluation by solid-state NMR technique and catalytic properties. J Phys Chem C 2015; 119: 15303–15. 10.1021/acs.jpcc.5b03289

[88] Wang Z , Chu W, Zhao Z et al. The role of organic and inorganic structure-directing agents in selective Al substitution of zeolite. J Phys Chem Lett 2021; 12: 9398–406. 10.1021/acs.jpclett.1c01448 34553943

[89] Devos J , Robijns S, Van Goethem C et al. Interzeolite conversion and the role of aluminum: toward generic principles of acid site genesis and distributions in ZSM-5 and SSZ-13. Chem Mater 2021; 7: 2516–31. 10.1021/acs.chemmater.0c04832

[90] Zhang J , Shan Y, Zhang L et al. Importance of controllable Al sites in CHA framework by crystallization pathways for NH_3_-SCR reaction. Appl Catal B 2020; 277: 119193. 10.1016/j.apcatb.2020.119193

[91] Huang X , Ma M, Li M et al. Regulating the location of framework aluminum in mordenite for the carbonylation of dimethyl ether. Catal Sci Technol 2020; 10: 7280–90. 10.1039/D0CY01362E

[92] Devos J , Bols M, Plessers D et al. Synthesis-structure-activity relations in Fe-CHA for C-H activation: control of Al distribution by interzeolite conversion. Chem Mater 2020; 32: 273–85. 10.1021/acs.chemmater.9b03738

[93] Wang S , Zhang L, Li S et al. Tuning the siting of aluminum in ZSM-11 zeolite and regulating its catalytic performance in the conversion of methanol to olefins. J Catal 2019; 377: 81–97. 10.1016/j.jcat.2019.07.028

[94] Biligetu T , Wang Y, Nishitoba T et al. Al distribution and catalytic performance of ZSM-5 zeolites synthesized with various alcohols. J Catal 2017; 353: 1–10. 10.1016/j.jcat.2017.06.026

[95] Muraoka K , Chaikittisilp W, Yanaba Y et al. Directing aluminum atoms into energetically favorable tetrahedral sites in a zeolite framework by using organic structure-directing agents. Angew Chem Int Ed 2018; 57: 3742–6. 10.1002/anie.201713308 29405535

[96] Chu W , Liu X, Yang Z et al. Constrained Al sites in FER-type zeolites. Chin J Catal 2021; 42: 2078–87. 10.1016/S1872-2067(21)63884-6

[97] Han S , Tang X, Wang L et al. Potassium-directed sustainable synthesis of new high silica small-pore zeolite with KFI structure (ZJM-7) as an efficient catalyst for NH_3_-SCR reaction. Appl Catal B 2021; 281: 119480. 10.1016/j.apcatb.2020.119480

[98] Zones S , Hwang S. The inorganic chemistry of guest-mediated zeolite crystallization: a comparison of the use of boron and aluminum as lattice-substituting components in the presence of a single guest molecule during zeolite synthesis. Microporous Mesoporous Mater 2003; 58: 263–77. 10.1016/S1387-1811(02)00653-4

[99] Zones S , Darton R, Morris R et al. Studies on the role of fluoride ion vs reaction concentration in zeolite synthesis. J Phys Chem B 2005; 109: 652–61. 10.1021/jp0402434 16851058

